# Baseline Functional Performance Predicts Better Long-Term Self-Reported Physical Function After Auto-HSCT

**DOI:** 10.3390/jcm15114318

**Published:** 2026-06-03

**Authors:** Lindsey J. Anderson, Lily Okamura, Nina Dhunjishah, Roshni Gowrisankar, Jennifer Song, Thomas R. Chauncey, Jose M. Garcia

**Affiliations:** 1 Geriatric Research, Education and Clinical Center, Veterans Affairs Puget Sound Health Care System, Seattle, WA 98108, USA; 2 Division of Gerontology and Geriatric Medicine, Department of Medicine, University of Washington, Seattle, WA 98195, USA; 3 Fred Hutchinson Cancer Research Center, Seattle, WA 98109, USA; 4 Bone Marrow Transplant Unit, Veterans Affairs Puget Sound Health Care System, Seattle, WA 98108, USA; 5 Oncology, Department of Medicine, University of Washington, Seattle, WA 98195, USA

**Keywords:** autologous hematopoietic stem cell transplantation, physical function, quality of life, muscle mass, muscle function, 30 s chair stand test, 6 min walk test

## Abstract

**Background/Objectives**: Determination of baseline predictors of longer-term quality of life (QOL) after autologous hematopoietic stem cell transplantation (Auto-HSCT) may identify patients with the greatest supportive care needs. We hypothesized that baseline older age, weight loss, and worse functional performance would negatively predict QOL over two years post-HSCT. **Methods**: Physical function, body composition, and QOL were assessed before (PRE) and one month (1MO) after Auto-HSCT in U.S. Veterans (*N* = 23). QOL and survival were also assessed approximately every six months for two years after Auto-HSCT (5MO, 1YR, 1.5YR, and 2YR). Changes over time were tested via Generalized Estimating Equation regression analyses (*p* < 0.05 = significant). The impact of PRE variables on QOL at each follow-up was tested via Spearman’s correlations (*p* < 0.01 = significant). **Results**: Relative to PRE, depression and anxiety significantly improved (*p* ≤ 0.039) at 1MO while fatigue and vitality significantly worsened (*p* ≤ 0.024) 1MO to 5MO post-HSCT. Vitality, depression, and anxiety returned to PRE levels thereafter, while fatigue trajectory varied depending on the survey used. Bone-Marrow-Transplant-related QOL significantly improved at 5MO (*p* = 0.014) while self-reported function (*p* ≤ 0.021) and physical activity (*p* ≤ 0.045) significantly improved 1-2YR post-HSCT. Greater PRE 30 s chair stand test performance consistently correlated with better self-reported function 1-2YR (*r* = 0.76–0.91, *p* ≤ 0.007) post-HSCT. Greater PRE 6 min walk test performance consistently correlated with better symptom burden 1-2YR (*r* = 0.71–0.81, *p* ≤ 0.01) post-HSCT. **Conclusions**: In support of our hypothesis, baseline functional performance was associated with QOL during two years of recovery after Auto-HSCT; older age and recent weight loss at baseline only predicted worse baseline QOL. Our data indicates that evaluation of the 30 s chair stand and 6 min walk tests as rehabilitation targets and/or predictors of QOL, fitness, or mortality after Auto-HSCT are warranted. Larger, controlled studies are needed to confirm the findings from this exploratory analysis.

## 1. Introduction

Hematopoietic stem cell transplant (HSCT) is a standard treatment to prolong disease-free and overall survival in patients with hematologic cancer. Roughly 90,000 HSCTs are performed worldwide annually to treat various hematologic malignancies, over 53% of which are autologous “Auto” procedures [1]. Though typically well tolerated with anticipated short-term toxicities, Auto-HSCT can result in significant morbidity, with chronic effects like functional impairment, pain, and fatigue being reported by some survivors for years afterward. The direct effects of malignancy, chemotherapy, radiation, drug treatment, and sedentary behavior during HSCT all likely contribute to increased fatigue and reduced overall quality of life (QOL) after Auto-HSCT [2]. Muscle strength is also lower in patients with hematologic malignancy (prior to HSCT) than age-related healthy controls [3], which further contributes to reduced QOL and physical activity after HSCT [2,4].

Baseline older age and worse patient-reported QOL negatively predict QOL one year after Auto-HSCT [5,6]. Older age, in addition to male sex, poor performance rating, greater comorbidity burden, and greater treatment exposure prior to Auto-HSCT are also risk factors for worse overall mortality one to three years after Auto-HSCT [7,8]. Of these risk factors, functional performance may have the greatest potential to be modifiable; however, a more detailed characterization of baseline phenotypes and performance measures that predict longer-term QOL after Auto-HSCT may identify patients with the greatest supportive care needs. The purpose of this study was to identify baseline factors impacting patient-reported QOL across two years after Auto-HSCT. We hypothesized that older age, weight loss, lower muscle mass, and worse functional performance prior to HSCT would be associated with worse QOL over two years of recovery post-HSCT.

## 2. Materials and Methods

### 2.1. Study Participants and Design

We have previously reported on short-term outcomes affecting patient fitness and/or contributing to development of cachexia for U.S. Military Veterans undergoing Auto-HSCT [9]. This secondary analysis aims to identify factors contributing to patient-reported QOL over two years after Auto-HSCT. There are only two Bone Marrow Transplant Units in the VA system, one in Seattle, WA, and one in Nashville, TN. Patients over 18 years of age planning Auto-HSCT at the Veterans Affairs Puget Sound Health Care System (VAPSHCS) Bone Marrow Transplant Unit in Seattle, WA, were included; patients who declined participation were excluded. Recruitment occurred between February 2017 and July 2019. The protocol was approved by the VAPSHCS Institutional Review Board and the Research and Development Committee. The protocol was conducted in compliance with the Declarations of Helsinki and its amendments and the International Conference on Harmonization Guidelines for Good Clinical Practices.

Study assessments occurred prior to chemomobilization (PRE), 30 ± 10 days after Auto-HSCT (Follow-Up 1; 1MO), and at roughly 6-month intervals for up to two years post-HSCT (5MO, 1YR, 1.5YR, and 2YR). For PRE and 1MO visits, patients reported to the VAPSHCS in the morning after fasting overnight for assessment of body composition, physical function, and patient-reported outcomes (PROs) and collection of a blood sample. Blood was collected in EDTA-containing vacutainers before 10 a.m.; vacutainers were centrifuged and the plasma layer was aliquoted for storage at −80 °C until analysis. For 5MO-2YR visits, patients completed PRO questionnaires via postal mail. The primary analysis previously reported data from the PRE and 1MO visits only [9]; herein we report data from PRE and all follow-up time points.

### 2.2. Body Composition

Dual-energy X-ray absorptiometry (Hologic Inc., Marlborough, MA, USA) was used to estimate appendicular lean mass, appendicular skeletal muscle index [appendicular lean mass (kg)/height (m^2^)], total fat mass, and percent body fat as previously described [9].

### 2.3. Physical Function

The 6-Minute Walk Test (6MWT), peak aerobic capacity (VO_2_Peak; Vmax Encore, Vyaire Medical, Inc.; Mettawa, IL, USA), hand grip strength (Jamar Hydraulic Dynamometer, J.A. Preston Corp., Downers Grove, IL, USA), stair climb power, one-repetition maximal muscle strength (knee extension, knee flexion, right hip extension, chest press, latissimus pull-down, and upper back seated row; Kaiser Sports Health Equipment, Inc., Fresno, CA, USA), and the 30 s chair stand test (30-CST) were administered as described previously [9].

### 2.4. Patient-Reported Outcomes

The Anderson Symptom Assessment Scale (ASAS) [10], Short Form-36 Health Survey (SF-36) [11], Functional Assessment of Chronic Illness Therapy-Fatigue (FACIT-F) [12], Functional Assessment of Cancer Therapy-Bone Marrow Transplant Scale (FACT-BMT) [13], Positive and Negative Affect Schedule (PANAS) [14], Global Assessment of Change Questionnaire (GCGQ) [15], Patient Health Questionnaire (PHQ-8) [16], and Rapid Assessment of Physical Activity (RAPA: Aerobic Exercise and Strength/Flexibility scores) [17] were assessed at all time points. The FACIT-F and FACT-BMT each contain four core modules, Functional, Social/Family, Emotional, and Physical Well-Being, in addition to a Fatigue or Transplant supplement, respectively. Except for PANAS-Negative Affect, all other PRO scores were converted as necessary so that larger numbers indicate better quality of life (QOL), and smaller numbers indicate worse QOL. These tests were administered as described previously [9].

### 2.5. Plasma Biomarkers

Inflammatory cytokines interleukin-6 (IL-6; pg/mL) and tumor necrosis factor (TNF; pg/mL) in plasma were detected by V-PLEX Human Pro-inflammatory Panel 1 from Meso Scale Discovery company (Cat# N05049 A-1, MSD, Rockville, MD, USA) as previously described [9]. Plasma sex hormones including 17-hydroxypregnenolone (ng/mL), 17-hydroxyprogesterone (ng/mL), androstenedione (ng/mL), androsterone (ng/mL), dehydroepiandrosterone (DHEA; ng/mL), dihydrotestosterone (DHT; ng/mL), pregnenolone (ng/mL), progesterone (ng/mL), and total testosterone (ng/mL) were analyzed via liquid-chromatography tandem mass spectroscopy while sex-hormone binding globulin (SHBG; nmol/L) was measured with the Quantikine SHBG Immunoassay (R&D Systems, Minneapolis, MN, USA) as previously reported [18].

### 2.6. Statistical Analysis

SPSSv30 (SPSS, Inc., Chicago, IL, USA) was used for statistical analysis; data are presented as mean (SEM) or N (%) unless otherwise noted. PRE-HSCT PRO scores were compared between patients with or without (1) weight loss (>5% body weight loss in the prior six months); (2) myeloma; (3) White, non-Hispanic background; (4) recent chemotherapy exposure; (5) or recent glucocorticoid exposure using Mann–Whitney independent samples U-tests to determine baseline covariates. Associations at PRE between PRO scores and age or time since diagnosis were also tested for baseline covariates using Spearman’s correlations. All other associations between PRE or follow-up time points were also performed using Spearman’s correlations.

Repeated measures were compared with Generalized Estimating Equation (GEE) regression analyses, modeling patients as a random effect and time as the primary predictor (ordinal variable coded as PRE, 1MO, 5MO, 1YR, 1.5YR, and 2YR). A robust covariance matrix with an exchangeable structure was used to account for correlation among repeated measurements for the same individual. If PRE PRO scores were significantly different between baseline covariate comparisons as described above, the GEE regression analysis for that PRO was adjusted for the covariate. RAPA Strength/Flexibility category is not strictly ordinal and was coded into a dichotomous variable for assessment of repeated measures via Fisher’s Exact test (0 = no strength or flexibility exercise reported; 1 = either strength or flexibility or both exercises reported).

Statistical significance testing was 2-tailed, with *p* < 0.05 considered significant for U-tests and GEE regression analyses; *p* < 0.01 was considered significant for Spearman’s correlations to reduce the Type 2 error rate from multiple comparisons.

## 3. Results

### 3.1. Study Participants

**Figure 1:**
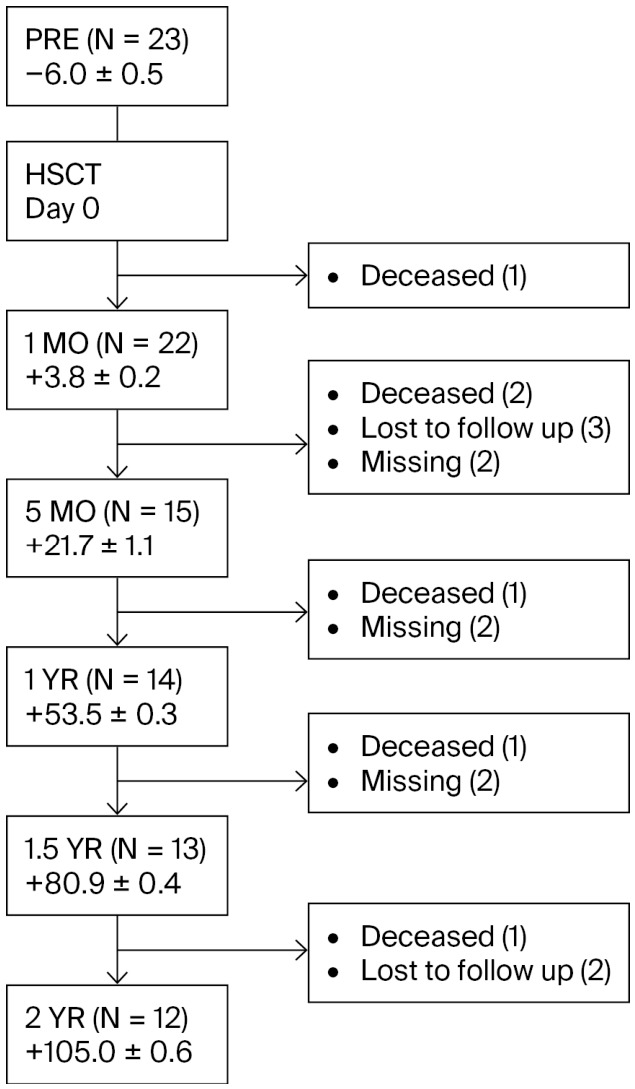
CONSORT diagram depicting the timing of visits relative to hematopoietic stem cell transplantation (HSCT = Day 0), where negative or positive days represent the number of weeks before or after transplant, respectively. PRE, baseline; N, sample size; MO, month; YR, years. Data are presented as mean ± standard deviation. The number of persons deceased, lost to follow-up, or with missing data are indicated in parentheses; persons were categorized as “missing” if they completed assessments at a later visit.

Participants were predominantly White, non-Hispanic males with multiple myeloma (Table 1). Roughly half were recently exposed to chemotherapy and/or glucocorticoid treatment prior to PRE and 26% experienced weight loss over the six months prior to PRE (Table 1). Visit timing relative to Auto-HSCT and sample sizes at each time point are provided in Figure 1. Overall, *N* = 6 (26.4%) deceased and *N* = 5 (21.7%) were lost to follow-up throughout the study. The conditioning regimens included melphalan for patients with multiple myeloma and BEAM/mini-BEAM (carmustine, etoposide, cyratabine, and melphalan) for those with lymphoma.

### 3.2. Baseline Covariates

Six out of eight patients with lymphoma displayed recurrence at PRE, while only one out of 15 with multiple myeloma displayed recurrence; therefore, we did not test for recurrence as a covariate independently from diagnosis. At PRE, SF-36 bodily pain was significantly worse in those with multiple myeloma than lymphoma (*p* = 0.016). PRE SF-36 total, GCGQ, PANAS-Negative Affect, FACT BMT supplement, and ASAS anxiety, appetite loss, sleeplessness, overall wellness, and total scores were significantly worse in patients with recent weight loss than those with stable weight (*p* ≤ 0.037). PRE PRO scores were not significantly different between those with or without White, non-Hispanic background or between those with or without recent chemotherapy or glucocorticoid exposure (*p* > 0.05). PRE PRO scores were not significantly correlated with age or time since diagnosis at PRE (*p* > 0.05). Therefore, GEE regression analyses were only adjusted for diagnosis and baseline weight loss as applicable.

### 3.3. Correlations Between PRE Variables

**Figure 2:**
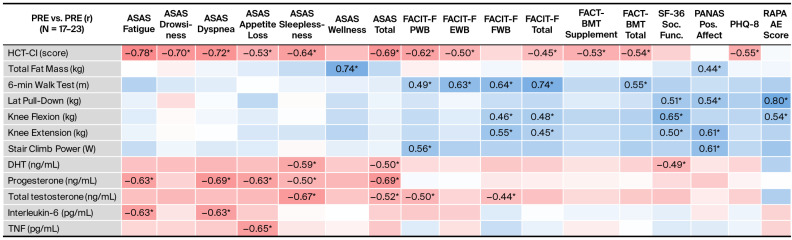
Summary heatmap of Spearman’s correlations between baseline variables. PRE, baseline; ASAS, Anderson Symptom Assessment Scale; FACIT-F, Functional Assessment of Chronic Illness Therapy-Fatigue; P/E/FWB, Physical/Emotional/Functional Well-Being; FACT-BMT, Functional Assessment of Cancer Therapy-Bone Marrow Transplant Scale; SF-36 Soc. Func., Short Form-36 Health Survey: Social Function; PANAS, Positive and Negative Affect Schedule; Pos., Positive; PHQ-8, Patient Health Questionnaire-8; RAPA AE, Rapid Assessment of Physical Activity: Aerobic Exercise; HCT-CI, hematopoietic cell transplant-comorbidity index; Lat, latissimus; DHT, Dihydrotestosterone; TNF, tumor necrosis factor. Higher patient-reported scores reflect better quality of life. * *p* < 0.01; blue = −1 (associated with worse quality of life), red = +1 (associated with better quality of life) correlation coefficient.

Older age correlated with lower chest press strength (*r* = −0.67, *p* = 0.005, *N* = 16) but did not correlate with any PROs at PRE (as discussed above in “Baseline covariates”). Figure 2 displays a heatmap of correlations at PRE between PROs with other variables (*p* < 0.01 only). Worse Comorbidity Index correlated with worse ASAS fatigue, ASAS drowsiness, ASAS dyspnea, ASAS sleeplessness, ASAS Total, FACIT-Physical Well-Being, FACT-BMT supplement, FACT-BMT Total, and PHQ-8 scores.

Greater 6MWT correlated with greater FACIT-Emotional Well-Being, FACIT-Functional Well-Being, and FACIT-F Total scores. Greater latissimus pull-down strength correlated with greater RAPA-Aerobic Exercise score. Greater knee extension strength and stair climb power each correlated with greater PANAS-Positive Affect. Greater knee flexion strength correlated with greater SF36-Social Function. Greater total fat mass and percent body fat each correlated with greater ASAS-Wellness. Body composition variables did not correlate with any PROs at PRE.

Greater DHT correlated with worse FACIT-Social/Family Well-Being, while greater DHEA correlated with greater FACIT-Physical Well-Being. Greater progesterone correlated with worse ASAS-Fatigue, Appetite Loss, and Total score. Greater total testosterone correlated with worse ASAS-Sleeplessness, while greater SHBG correlated with worse FACIT-Emotional Well-Being. Greater IL-6 correlated with worse ASAS-Fatigue and Dyspnea, while greater TNF correlated with worse ASAS-Appetite Loss. Representative scatterplots of significant baseline correlations are displayed in Supplemental Figure S1.

### 3.4. PRO Changes Across Two Years

**Figure 3:**
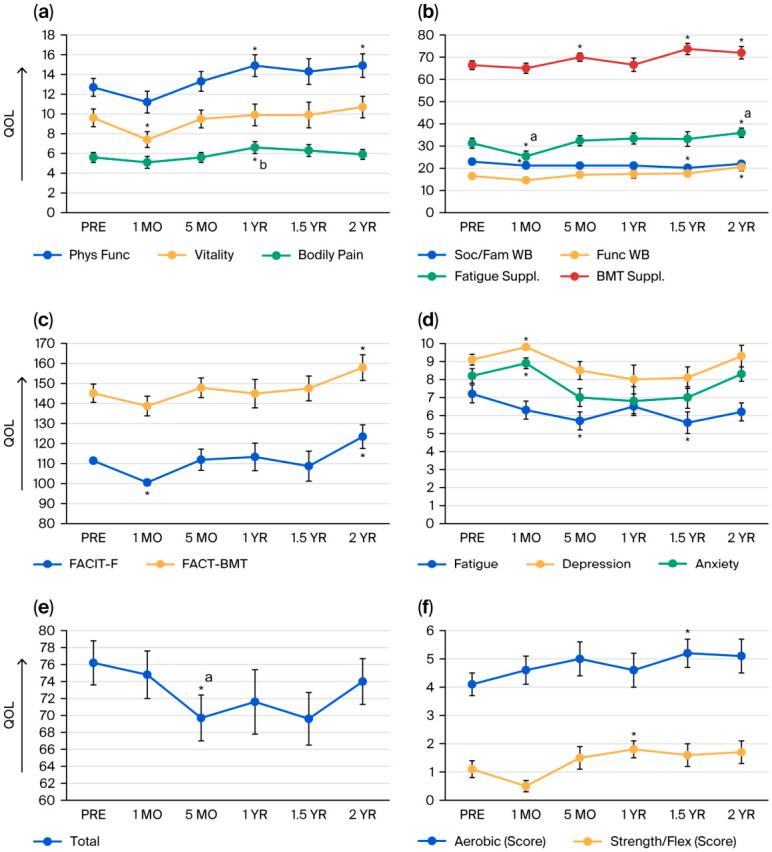
GEE regression comparison of selected patient-reported outcomes before (PRE) and after (1MO to 2YR) Auto-HSCT. Greater scores reflect better quality of life (QOL; indicated by arrow, lefthand side) for all of the components displayed here: (**a**) individual components of the Short Form-36; (**b**) individual components of the Functional Assessment of Chronic Illness Therapy-Fatigue “FACIT-F” or Functional Assessment of Cancer Therapy-Bone Marrow Transplant Scale “FACT-BMT”; (**c**) total scores for the FACIT-F or FACT-BMT; (**d**) individual components of the Anderson Symptom Assessment Scale “ASAS”; (**e**) total scores for the ASAS; or (**f**) individual components of the Rapid Assessment of Physical Activity. Phys Func, physical function; Soc/Fam WB; Social/Family Well-Being; Flex, flexibility exercise. * *p* < 0.05 vs. PRE; ^a^ adjusted for weight loss at PRE; ^b^ adjusted for diagnosis.

At 1MO, SF-36 vitality (Figure 3a) and FACIT-F/FACT-BMT Social/Family Well-Being, fatigue supplement (adjusted for weight loss at PRE), and FACIT-F/FACT-BMT Total scores significantly worsened (Figure 3b,c) relative to PRE. ASAS depression and anxiety significantly improved at 1MO relative to PRE (Figure 3d).

At 5MO, the BMT supplement score significantly improved (Figure 3b) while ASAS fatigue and Total (adjusted for weight loss at PRE) significantly worsened (Figure 3d,e) relative to PRE.

At 1YR, SF-36 Physical Function and Bodily Pain (adjusted for diagnosis; Figure 3a), GCGQ (adjusted for weight loss at PRE; *p* = 0.024), and RAPA Strength/Flexibility score (Figure 3f) significantly improved relative to PRE.

At 1.5YR, FACIT-F/FACT-BMT Social/Family Well-Being (Figure 3b) and ASAS fatigue (Figure 3d) significantly worsened relative to PRE. The BMT supplement score (Figure 3b) and RAPA-Aerobic Exercise score significantly improved relative to PRE (Figure 3f).

At 2YR, SF-36 Physical Function (Figure 3a) and FACIT-F/FACT-BMT Functional Well-Being (Figure 3b), fatigue supplement score (adjusted for weight loss at PRE), FACT-BMT supplement score (Figure 3b), and FACIT-F and FACT-BMT Total scores (Figure 3c) significantly improved relative to PRE.

There were no significant differences across time points for SF-36 Social Function, Mental Health, Role Limitations due to Physical Problems, Role Limitations due to Emotional Problems, or General Health Perception, for FACIT-F/FACT-BMT Emotional or Physical Well-Being, for PANAS Positive or Negative Affect, for PHQ-8 score, or for ASAS pain, nausea, drowsiness, breathlessness, appetite loss (adjusted for weight loss at PRE), sleeplessness (adjusted for weight loss at PRE), or overall wellness (*p* > 0.05).

### 3.5. Correlations Between Objective Outcomes at PRE with PROs Assessed at 1MO

Greater PRE percent body fat correlated with worse FACIT-Physical Well-Being (*r* = −0.58, *p* = 0.008, *N* = 20) at 1MO. Greater PRE mean hand grip strength (*r* = 0.63, *p* = 0.003, *N* = 20) and dominant hand grip strength (*r* = 0.59, *p* = 0.008, *N* = 19) each correlated with better PANAS-Positive Affect at 1MO. No other PRE body composition or physical function outcomes significantly correlated with PROs at 1MO (*p* > 0.01). Greater PRE DHT correlated with worse FACIT-Social/Family Well-Being (*r* = −0.63, *p* = 0.002, *N* = 21) and greater PRE IL-6 correlated with more severe ASAS-Appetite Loss (*r* = −0.66, *p* = 0.006, *N* = 16).

### 3.6. Correlations Between Objective Outcomes at PRE with PROs Assessed at 5MO

**Figure 4:**
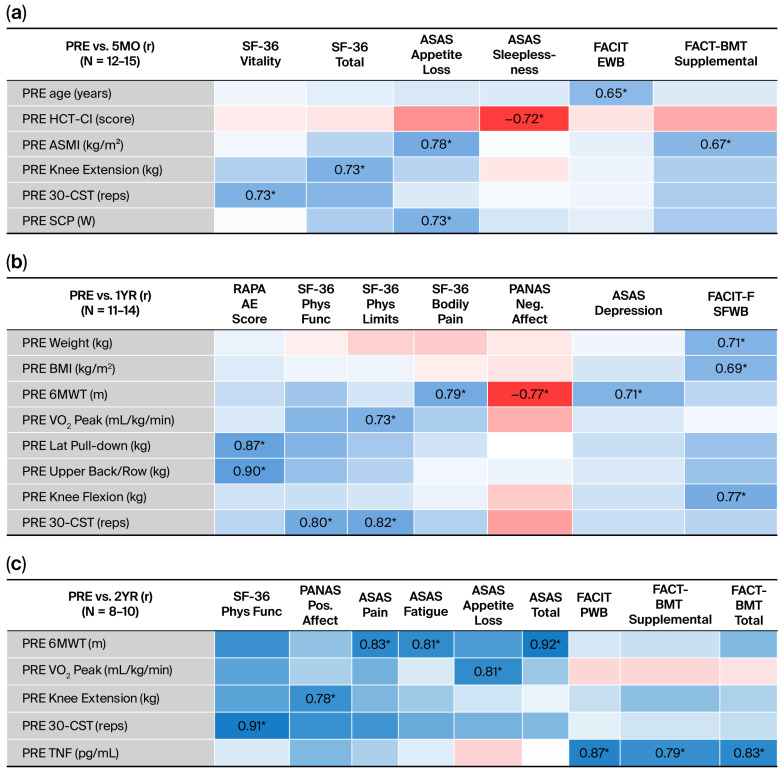
Summary heatmap of Spearman’s correlations between baseline (PRE) variables with patient-reported outcomes at (**a**) five months “5MO”; (**b**) one year “1YR”; or (**c**) two years “2YR” after transplantation. HCT-CI, hematopoietic cell transplant-comorbidity index; ASMI, appendicular skeletal muscle index; 30-CST, 30 s chair stand test; SCP, stair climb power; SF-36, Short Form-36; FACIT-F, Functional Assessment of Chronic Illness Therapy-Fatigue; EWB, Emotional Well-Being; FACT-BMT, Functional Assessment of Cancer Therapy-Bone Marrow Transplant Scale; BMI, body mass index; 6MWT, 6 min walk test; VO_2_Peak, peak aerobic capacity; RAPA AE, Rapid Assessment of Physical Activity: Aerobic Exercise; Phys Func, physical function; Phys Limits, physical limitations; PANAS, Positive and Negative Affect Schedule; Neg./Pos., Negative/Positive; SFWB, Social/Family Well-Being; TNF, tumor necrosis factor; ASAS, Anderson Symptom Assessment Scale; PWB, Physical Well-Being. Higher patient-reported scores reflect better quality of life. * *p* < 0.01; blue = −1 (associated with worse quality of life), red = +1 (associated with better quality of life) correlation coefficient.

Figure 4a displays a summary heatmap of correlations between objective outcomes at PRE with PRO at 5MO (*p* < 0.01 only). Greater age at PRE correlated with better Emotional Well-Being at 5MO, while greater PRE Comorbidity Index correlated with worse ASAS sleeplessness at 5MO. PRE appendicular skeletal muscle index and PRE stair climb power each correlated with better ASAS appetite at 5MO; greater PRE appendicular skeletal muscle index also correlated with better FACT-BMT supplement at 5MO. Greater PRE 30-CST correlated with better SF-36 vitality score at 5MO, and greater PRE knee extension strength correlated with better SF-36 total at 5MO. No other PRE body composition or physical function outcomes, nor any PRE biomarker levels, significantly correlated with PROs at 5MO (*p* > 0.01). Representative scatterplots are displayed in Supplemental Figure S2.

### 3.7. Correlations Between Objective Outcomes at PRE with PROs Assessed at 1YR

Figure 4b displays a summary heatmap of correlations between objective outcomes at PRE with PRO at 1YR (*p* < 0.01 only). Greater PRE weight, body mass index, and knee flexion strength each correlated with greater Social/Family Well-Being at 1YR. Greater PRE 6MWT correlated with better SF-36 Bodily Pain, PANAS-Negative Affect, and ASAS depression scores at 1YR. Greater PRE VO_2_Peak and PRE 30-CST correlated with less SF-36 Role Limitations due to Physical Problems at 1YR; greater PRE 30-CST also correlated with better SF-36 Physical Function score at 1YR. Greater PRE latissimus pull-down and upper back seated row strength correlated with greater RAPA-Aerobic Exercise score at 1YR. No other PRE body composition or physical function outcomes, nor any PRE biomarker levels, significantly correlated with PROs at 1YR (*p* > 0.01). Representative scatterplots are displayed in Supplemental Figure S3.

### 3.8. Correlations Between Objective Outcomes at PRE with PROs Assessed at 1.5YR

Greater PRE knee extension strength correlated with better SF-36 General Health Perception (*r* = 0.73, *p* = 0.005, *N* = 13) at 1.5YR and greater PRE 30-CST correlated with better SF-36 Physical Function (*r* = 0.76, *p* = 0.007, *N* = 11) at 1.5YR. No other PRE body composition or physical function outcomes, nor any PRE biomarker levels, significantly correlated with PROs at 1.5YR (*p* > 0.01). Representative scatterplots are displayed in Supplemental Figure S4.

### 3.9. Correlations Between Objective Outcomes at PRE with PROs Assessed at 2YR

Figure 4c displays a summary heatmap of correlations between objective outcomes at PRE with PRO at 2YR (*p* < 0.01 only). Greater PRE 6MWT correlated with better ASAS pain, fatigue, and total scores at 2YR. Greater PRE VO_2_Peak correlated with better ASAS appetite at 2YR, while greater PRE 30-CST correlated with better SF-36 Physical Function, and greater PRE knee extension strength correlated with better PANAS-Positive Affect at 2YR. Greater PRE TNF correlated with better FACIT Physical Well-Being, FACT BMT supplement, and FACT-BMT total scores (Figure 4c). Representative scatterplots are displayed in Supplemental Figure S5. No other PRE body composition, physical function, or biomarker outcomes significantly correlated with PROs at 2YR (*p* > 0.01).

### 3.10. Survival

Pre-HSCT weight, BMI, fat mass, RAPA-Aerobic activity score, Positive Affect, GCGQ score, and ASAS-Wellness all correlated with longer survival; however, these were nominally significant (*p* < 0.05) and did not reach significance at our pre-determined level (*p* < 0.01). No measures of objective physical function correlated with survival.

## 4. Discussion

In U.S. Veterans undergoing Auto-HSCT, we observed acute symptom fluctuation within five months post-HSCT, while self-reported physical function and physical activity took one to two years to improve above pre-HSCT levels, and better pre-HSCT physical function correlated with better self-reported functional performance one to two years post-HSCT. We hypothesized that pre-HSCT older age, recent weight loss, and worse functional performance would be negatively associated with QOL over two years post-HSCT. In support of this hypothesis, functional performance was the primary pre-HSCT factor associated with QOL during two years of recovery after Auto-HSCT, while older age and history of weight loss prior to HSCT only predicted worse baseline QOL. Our data indicates that evaluation of functional performance as a rehabilitation target and/or predictor of QOL, fitness, or mortality after Auto-HSCT is warranted. Larger, controlled studies are still needed to confirm the findings from this exploratory analysis.

Even though fluctuations in QOL have been previously reported after Auto-HSCT, the significance of baseline factors impacting these changes is not well understood and has not been examined in U.S. Veterans. Here we observed that fatigue increased while depression and anxiety decreased within five months post-HSCT, likely due to the physical impact of Auto-HSCT along with the relief of having completed the treatment; these symptoms generally returned to pre-HSCT levels thereafter. This is similar to another report where patient-reported QOL decreased acutely and returned to pre-HSCT levels between nine months and one year after allogeneic or Auto-HSCT [19]. However, QOL worsened again by one to two years post-HSCT in that study [19], while we did not observe a secondary worsening of QOL one to two years after Auto-HSCT in the current study. We only included Auto-HSCT here, but the type of transplant reportedly did not impact their findings; however, our cohort is almost a decade older on average. In contrast to our findings, a systematic review revealed that QOL and symptom scores were regained as early as 1–2 months post-HSCT in patients treated for multiple myeloma [20]. Another study that evaluated QOL trajectory after Auto-HSCT for multiple myeloma reported that FACT-BMT total score improved by one year post-HSCT [6], but we did not observe FACT-BMT improvement until two years post-HSCT in our current study. This discrepancy between these observations and the current findings may be due to the older average age of our cohort or to our inclusion of both multiple myeloma and lymphoma, but an improvement in QOL and pain was also noted on long-term follow-up in some studies. These observations portray the variability in symptom burden trajectory and recovery for HSCT recipients.

Our analysis revealed distinctive patterns with baseline physical function predicting physical function and symptom burden, respectively, one to two years post-HSCT. Greater pre-HSCT 30-CST was associated with fewer functional problems and with better self-reported function one to two years post-HSCT. This test also had predictive value on vitality five months post-HSCT, suggesting that this assessment may inform patients, caregivers, and the clinical care team and may serve as a target to improve with rehabilitation/exercise before, during, and/or after Auto-HSCT. Greater pre-HSCT 6MWT was associated with less self-reported bodily pain, negative affect/mood, depression, and fatigue one to two years post-HSCT. Greater pre-HSCT aerobic capacity was associated with fewer functional limitations one-year post-HSCT and with better appetite two years post-HSCT. As poor functional status is known to be associated with greater mortality risk after HSCT [21,22,23,24], our observations suggest that pre-HSCT physical function is an important contributor to QOL after Auto-HSCT. The 30-CST and 6MWT may be especially relevant as they predicted distinct domains of QOL here, and they are both easy tests to perform in the clinic.

It has been previously reported that lower baseline hand grip strength negatively predicts patient-reported QOL one-year after allogeneic HSCT [25], but we did not observe that relationship here. In terms of muscle strength, we observed that greater pre-HSCT knee extension (quadriceps) strength correlated with better QOL between five months and two years post-HSCT. This is important because 66% of patients fall below the age/sex-stratified 50th percentile for leg strength prior to HSCT [26]. Furthermore, older age, lower body mass index, and higher comorbidity burden are key determinants of lower strength prior to HSCT [26]. These observations highlight the importance of understanding the contribution of physical function to QOL after HSCT, especially in high-risk patients.

Our observations also imply that patients exhibiting worse symptom burden or poor physical function/functional rating may benefit from prehabilitation before Auto-HSCT. This may particularly benefit patients with multiple myeloma who reported greater bodily pain prior to HSCT here. Recent guidelines for older adults living with cancer recommend exercise to enhance various aspects of physical function, including strength and endurance [27]. The Auto-HSCT process begins roughly 6–8 weeks prior to transplantation, and patients are typically discharged from the transplant unit 3–4 weeks after HSCT; this time frame is considered the peri-HSCT period. To date, research studies described as “prehabilitation” in the HSCT setting were initiated during this peri-HSCT period. These studies have reported low-to-moderate benefits on physical function and patient-reported outcomes between 30 and 100 days post-HSCT [28,29,30]. They have not evaluated longer-term benefits on function or QOL. Moreover, completion of prehabilitation prior to the peri-HSCT period may improve transplant candidacy and risk profile and improve long-term functional and QOL outcomes after Auto-HSCT. Future studies should test whether improvement of 30-CST, 6MWT, or quadriceps strength prior to Auto-HSCT improves physical function and QOL outcomes long term post-HSCT.

The average age of adult HSCT recipients is over 60 years [31], and patients aged 60 or over display worse HSCT tolerance and greater mortality than middle-aged or younger patients [32]. This is especially relevant for U.S. Military Veterans who are older and predominantly male and who experience worse comorbidity burden than the general population [33]. In the current study, we observed that pre-HSCT older age correlated with lower chest press strength prior to Auto-HSCT but was not associated with QOL across two years of recovery after Auto-HSCT. This is in contrast to a report with allogeneic HSCT recipients where older age prior to HSCT negatively predicted QOL one-year after HSCT [25]. The discrepancy with the current analysis may be due to the different transplant type, older average age and/or limited age range, poorer fitness levels, and/or predominantly male sex in the current cohort, considering that U.S. Veterans display a larger proportion of men and greater comorbidity burden than the general population [34]. Recent reports suggest that age is only a predictor of poor HSCT tolerance in physically fit patients and that poor functional status is an independent risk factor for worse HSCT tolerance and worse survival 1–3 years post-HSCT [35,36,37]. This finding is supported by our current observations that better pre-HSCT functional performance was predictive of better post-HSCT QOL.

We previously reported that weight loss prior to Auto-HSCT negatively predicted functional performance one month after Auto-HSCT in Veterans [9]. Here, we hypothesized that history of recent weight loss at baseline would predict worse QOL after Auto-HSCT; however, it was only associated with worse QOL at baseline. Instead, greater pre-HSCT appendicular skeletal muscle index correlated with better appetite and Bone-Marrow-Transplant-related QOL five months post-HSCT. This supports prior reports that sarcopenia (low muscle mass and strength with aging) is associated with worse treatment tolerance and shorter survival in HSCT recipients [38]. Up to half of adult HSCT recipients exhibit sarcopenia within two years post-HSCT [39]. It is also suggested that sarcopenic obesity (sarcopenia with excess body fat) increases the risk of morbidity and mortality during and up to five years after HSCT when compared with obesity or sarcopenia alone [40,41,42,43]. While we were unable to assess body composition beyond one month post-HSCT, we did observe that greater pre-HSCT body fat percentage predicted worse physical well-being one month post-HSCT, but not later. These findings are consistent with existing literature suggesting that body composition may be an important contributor to patients’ risk profile during HSCT. Notably, biomarkers tested here did not consistently predict changes in QOL up to 2 years post-HSCT, highlighting the importance of careful clinical assessment pre-HSCT in this population.

The main limitation of our study is that the design does not allow us to ascertain causality, and more studies are needed to test if improving baseline physical function improves QOL long term after Auto-HSCT. In addition, the original study was not designed to delineate differences between myeloma and lymphoma and was not statistically powered to do so. Instead, we chose to evaluate diagnosis as a baseline covariate and adjust regression analyses accordingly. Moreover, Veterans returned to their local treatment centers across the U.S. immediately after the one-month follow-up, which impeded retention for QOL across follow-up time points and prevented physical function and body composition assessment after the one-month follow-up. Recruitment for this study was completed prior to the COVID-19 pandemic, wherein remote assessment of physical function became feasible and validated. However, use of GEE in this study to assess QOL over time allowed for full utilization of missing data without requiring imputation, which can be especially problematic in smaller sample sizes. Our findings are most applicable to U.S. Veterans, particularly older Veterans with multiple myeloma, undergoing HSCT. More studies are needed to test whether these findings are also generalizable to non-Veterans and/or younger or middle-aged adults. However, the spectrum of fitness/performance categories tested, assessment of body composition via gold-standard methodology, and validated PRO surveys are major strengths of our design, which contribute to our confidence in our observations reported here.

The observations in the current study highlight the importance of physical function prior to HSCT, especially in older patients. However, 32.1% of health care providers reported that patients were “rarely or never” given physical activity advice as part of routine practice prior to HSCT, and another 25.4% reported that patients were only given physical activity advice “sometimes” [44]. Most (76.9%) health care providers also reported that physical activity was “rarely or never” measured prior to HSCT by questionnaire or physical assessment [44]. Our findings indicate that adverse effects on physical function and activity levels after Auto-HSCT may persist longer than fatigue and that patients with better pre-HSCT physical function display better QOL one to two years post-HSCT. The findings in this exploratory analysis suggest that future studies focusing on prehabilitation of patients planning Auto-HSCT, especially those with poor functional status, are warranted. Future studies may also consider evaluation of 30-CST and 6WMT performance as rehabilitation targets and/or predictors of QOL, fitness, and mortality after Auto-HSCT. Functional outcomes could be evaluated in person or remotely 2–5 years post-transplant for confirmation and expansion of the current findings. We also suggest that sufficient sample size should be included to differentiate effects between those with multiple myeloma and lymphoma and that each of these patient groups should be compared to a non-cancer, age-related control group.

## Figures and Tables

**Table 1:** Baseline descriptives and visit timing relative to transplantation.

Mean ± SEM or *N* (%)	*N* = 23
Age (year)	62.3 ± 2.4
Ht (cm)	174.9 ± 1.9
Wt (kg)	91.3 ± 3.8
BMI (kg/m^2^)	29.7 ± 1.0
Male	21 (91.3)
Ethnicity	
White non-Hispanic	15 (65.2)
White Hispanic	1 (4.3)
African American	3 (13.0)
Asian/Pacific Islander	2 (8.7)
Mixed	2 (8.7)
Diagnosis	
Multiple Myeloma	15 (65.2)
Lymphoma ^a^	8 (34.8)
Weeks since diagnosis/recurrence	48.3 ± 10.4
Recurrence (y)	7 (30.4)
Recent chemotherapy exposure (y) ^b^	12 (52.2)
Recent glucocorticoid exposure (y) ^c^	12 (52.2)
Recent weight loss (y) ^d^	6 (26.1)

^a^ Including Diffuse Large B-Cell, Mantle Cell, Non-Hodgkin, Hodgkin, and Follicular Lymphoma; ^b^ within 1-month before PRE; ^c^ within 3 months before PRE; ^d^ >5% weight loss within 6 months before PRE; BMI, body mass index; HCT-CI, hematopoietic cell transplant-comorbidity index; y, yes.

## Data Availability

The datasets presented in this article are not readily available because of Department of Veterans Affairs privacy policies. Requests to access the datasets should be directed to the corresponding author.

## Supplementary Materials

The following supporting information can be downloaded at: https://www.mdpi.com/article/10.3390/jcm15114318/s1, Figure S1: Selected Baseline Correlations; Figure S2: PRE functional performance predicts quality of life 5-Months post-HSCT; Figure S3: PRE functional performance correlates with quality of life 1-Year post-HSCT; Figure S4: PRE functional performance correlates with quality of life 1.5-Years post-HSCT; Figure S5: PRE functional performance correlates with quality of life 2-Years post-HSCT.

## Abbreviations

The following abbreviations are used in this manuscript:

Auto-HSCT	Autologous hematopoietic stem cell transplantation
QOL	quality of life
VAPSHCS	Veterans Affairs Puget Sound Health Care System
PRO	patient-reported outcomes
PRE	Baseline; prior to chemomobilization
1MO	One month
5MO	Five months
1YR	One year
1.5YR	1.5 years
2YR	Two years
6MWT	6-Minute Walk Test
VO_2_Peak	peak aerobic capacity
30-CST	30-second chair stand test
ASAS	Anderson Symptom Assessment Scale
FACIT-F	Functional Assessment of Chronic Illness Therapy-Fatigue
FACT-BMT	Functional Assessment of Cancer Therapy-Bone Marrow Transplant Scale
PANAS	Positive and Negative Affect Schedule
GCGQ	Global Assessment of Change Questionnaire
RAPA	Rapid Assessment of Physical Activity
IL-6	interleukin-6
TNF	tumor necrosis factor
DHEA	dehydroepiandrosterone
DHT	dihydrotestosterone
GEE	Generalized Estimating Equation
