# Supplementary material for: Baseline Functional Performance Predicts Better Long-Term Self-Reported Physical Function After Auto-HSCT
Source: J Clin Med. 2026 Jun 3;15(11):4318. doi: 10.3390/jcm15114318 (PMC13257520; doi:10.3390/jcm15114318)
Supplement: Supplementary file 1 [file jcm-15-04318-s001.zip › jcm-4310634-supplementary.pdf]

Supplemental Figure 1 (a-b). Selected Baseline Correlations

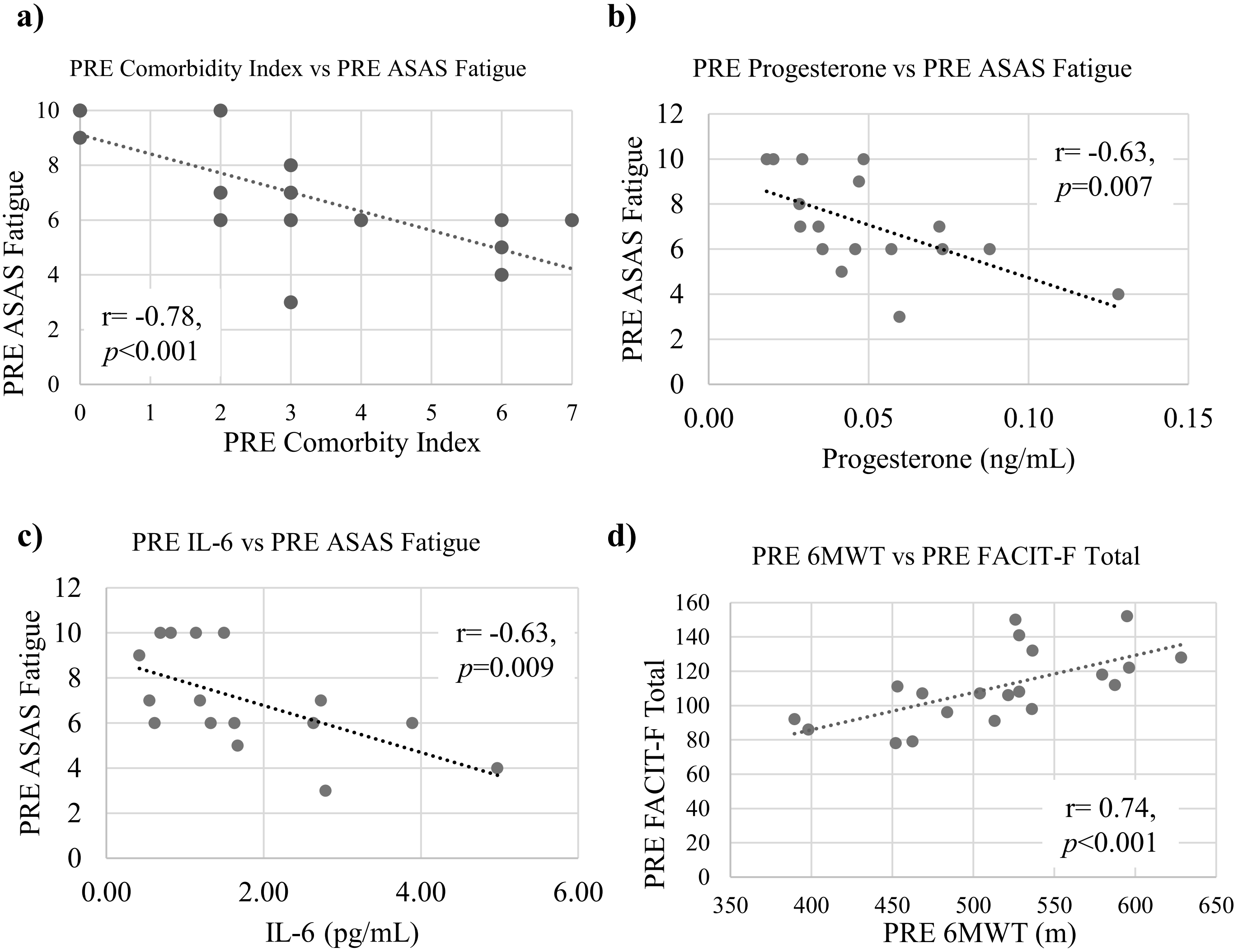

Scatterplots of Spearman's correlation between baseline (PRE) Anderson Symptom Assessment Scale (ASAS) Fatigue score versus PRE hematopoietic cell transplant-comorbidity index (a), PRE progesterone (b), or PRE interleukin (IL)-6 (c) or between PRE 6-minute walk test (6MWT) performance versus PRE Functional Assessment of Chronic Illness Therapy-Fatigue (FACIT-F) total score (d). Larger scores for the ASAS and FACIT-F surveys indicate better quality of life outcomes. Panels a-c indicate that more co-morbidities and higher progesterone and IL-6 levels are associated with more severe fatigue, while better 6MWT performance is associated with better overall quality of life at PRE.

Supplemental Figure 2 (a-b). PRE functional performance predicts quality of life 5-Months post-HSCT

a)

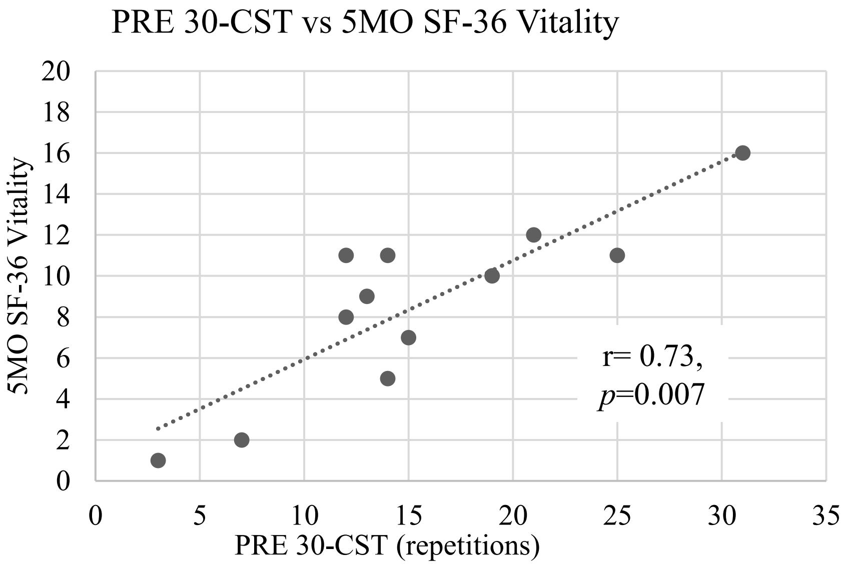

b)

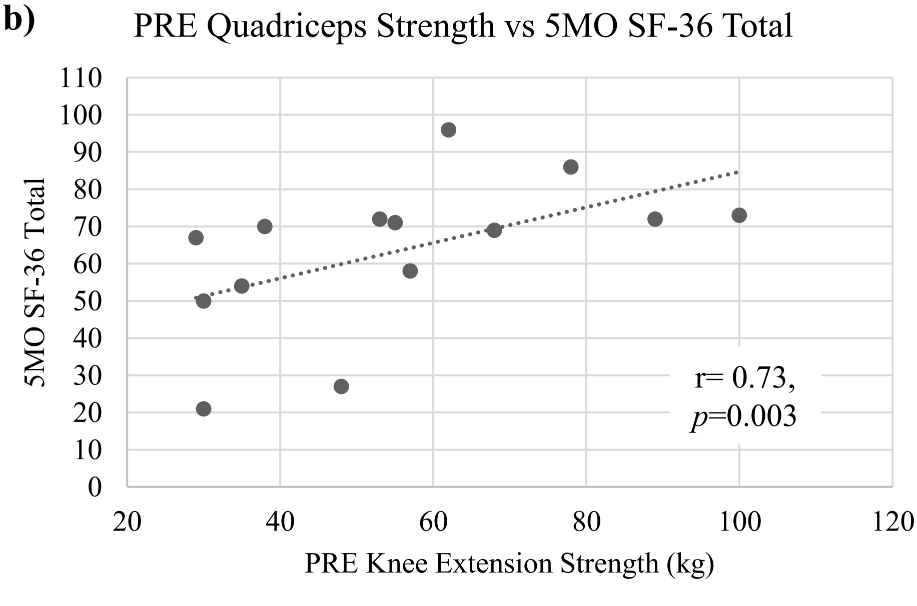

Scatterplots of Spearman's correlation between baseline (PRE) 30-second chair stand test (30-CST) performance versus Short Form-36 Health Survey (SF-36) Vitality score measured five months (5MO) after hematopoietic stem cell transplant (HSCT) (a) or between PRE knee extension strength versus SF-36 Total score measured at 5MO (b).

Supplemental Figure 3 (a-e). PRE functional performance correlates with quality of life 1-Year post-HSCT

a)

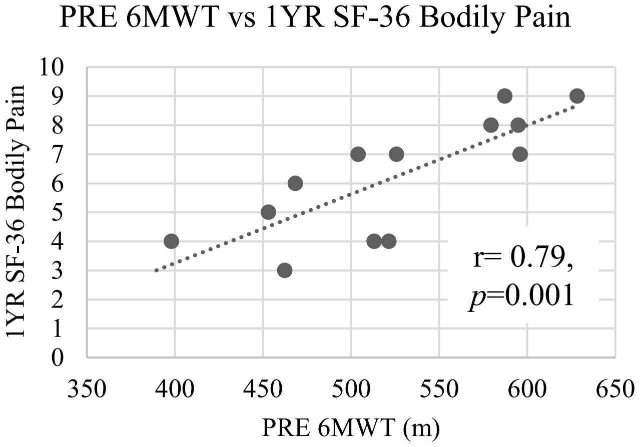

b)

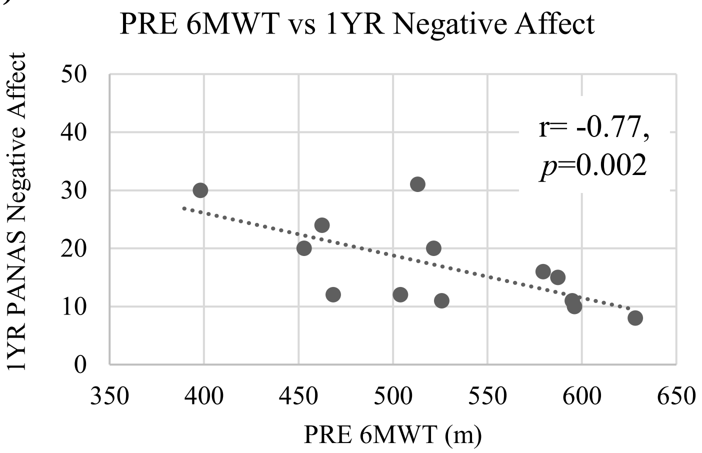

c)

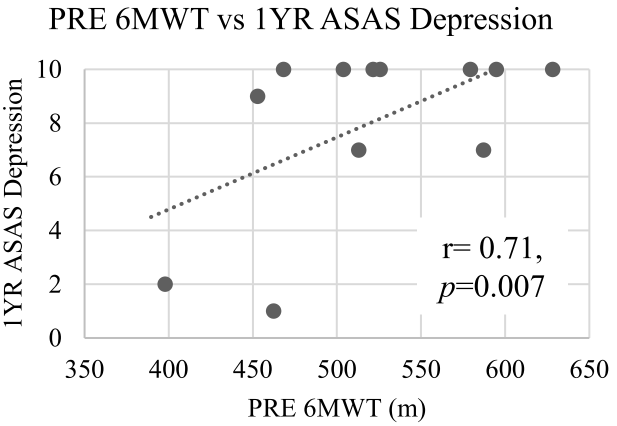

d)

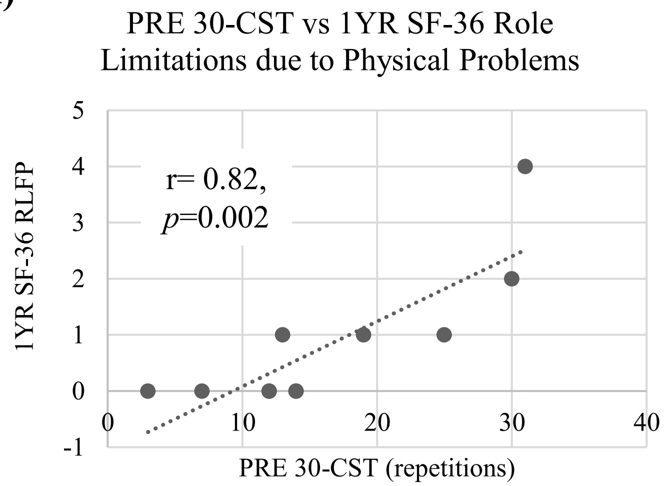

e)

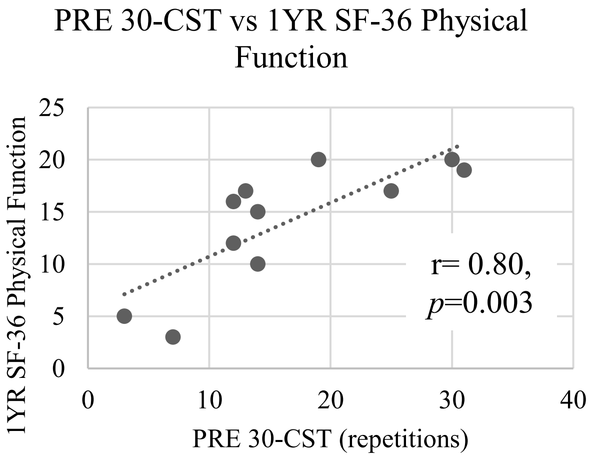

Scatterplots of Spearman's correlation between baseline (PRE) 6-minute walk test (6MWT) performance versus Short Form-36 Health Survey (SF-36) Bodily Pain (a), Positive and Negative Affect Schedule Negative Affect (b), and Anderson Symptom Assessment Scale (ASAS) Depression score (c) measured one year (1YR) after hematopoietic stem cell transplant (HSCT) or between PRE 30-second chair stand test (30-CST) performance versus SF-36 Role Limitations due to Physical Problems (d) or SF-36 Physical Function score (e).

Supplemental Figure 4 (a-b). PRE functional performance correlates with quality of life 1.5-Years post-HSCT

a)

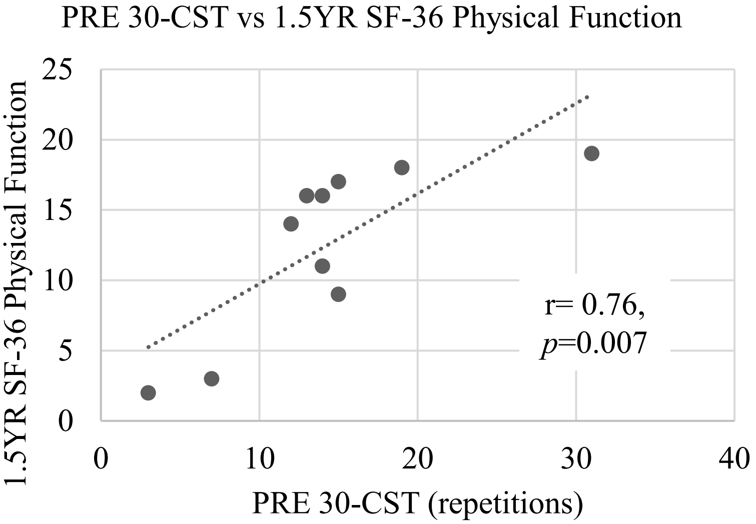

b)

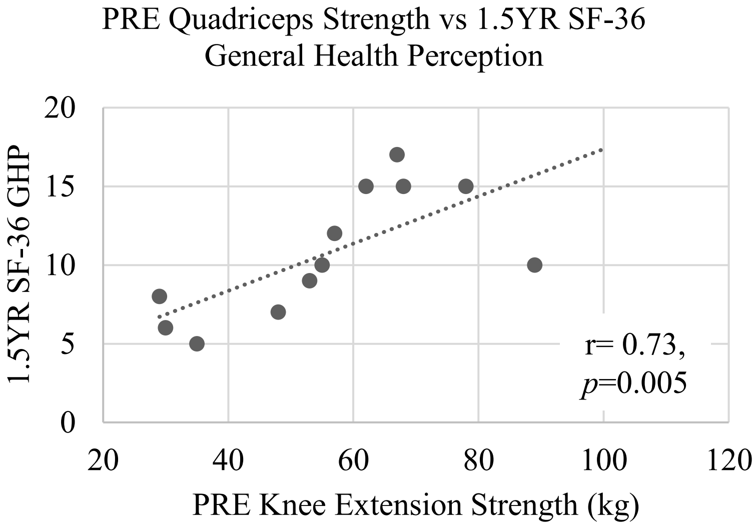

Scatterplots of Spearman's correlation between baseline (PRE) 30-second chair stand test (30-CST) performance versus Short Form-36 Health Survey (SF-36) Physical Function score measured 1.5 years (1.5YR) after hematopoietic stem cell transplant (HSCT) (a) or between PRE knee extension strength versus SF-36 General Health Perception score measured at 1.5YR (b).

Supplemental Figure 5 (a-e). PRE functional performance correlates with quality of life 2-Years post-HSCT

a)

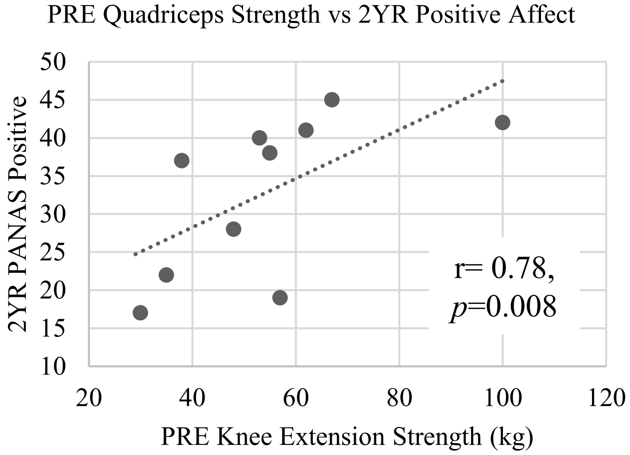

b)

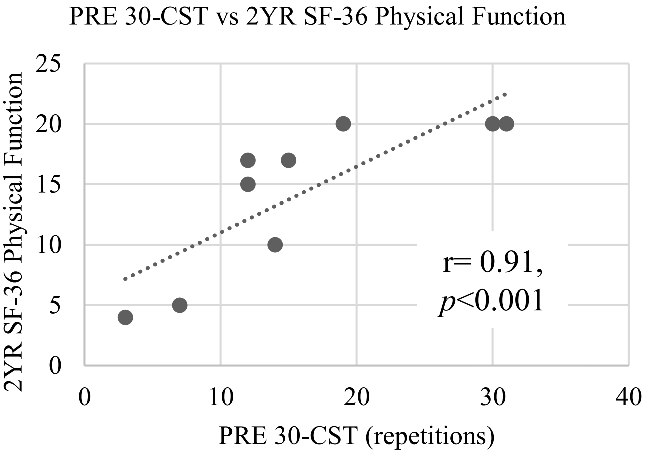

c)

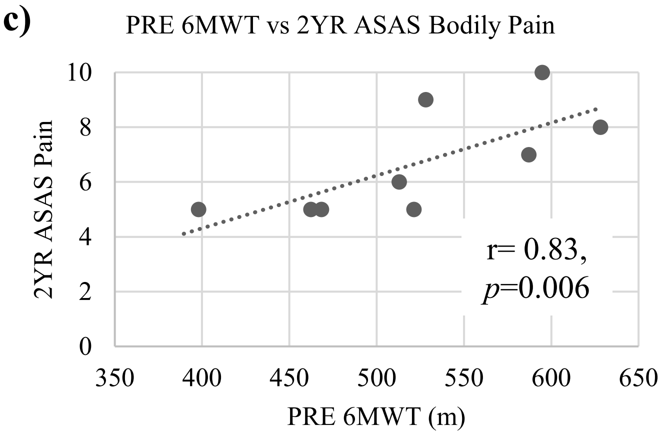

d)

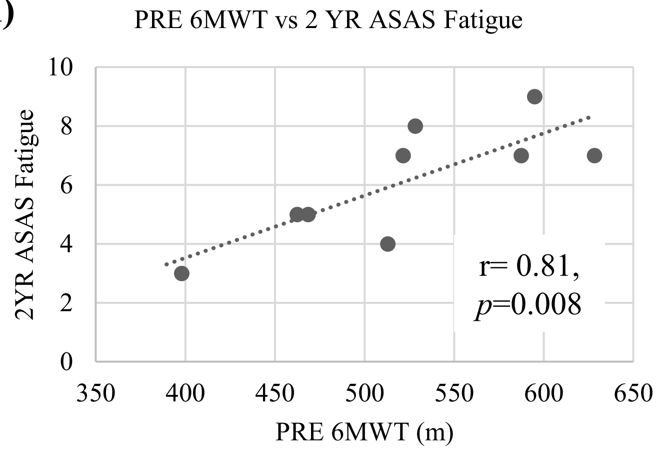

e)

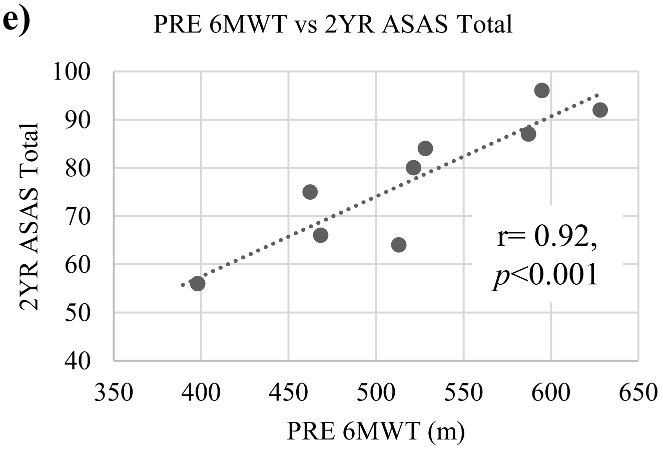

Scatterplots of Spearman's correlations between baseline (PRE) knee extension strength versus Positive and Negative Affect Schedule (PANAS) Positive Affect measured two years (2YR) after hematopoietic stem cell transplant (HSCT) (a), between PRE 30-second chair stand test (30-CST) performance versus Short Form-36 Health Survey (SF-36) Physical Function score at 2YR (b), or between PRE 6-minute walk test (6MWT) performance versus Anderson Symptom Assessment Scale (ASAS) Bodily Pain (c), ASAS Fatigue (d), or ASAS Total score (e) at 2YR. Larger scores for these surveys indicate better quality of life outcomes. Panels c-d indicate that better 6MWT performance at PRE is associated with less pain and fatigue 2YR post-HSCT.
